# Association between childhood trauma, cognition, and psychosocial function in a large sample of partially or fully remitted patients with bipolar disorder and healthy participants

**DOI:** 10.1186/s40345-023-00311-w

**Published:** 2023-09-20

**Authors:** Kamilla Woznica Miskowiak, Katrine Bang Hansen, Johanna Mariegaard, Lars Vedel Kessing

**Affiliations:** 1 Neurocognition and Emotion in Affective Disorders (NEAD) Centre, Mental Health Services, Capital Region of Denmark, and Department of Psychology, University of Copenhagen, Øster Farimagsgade 2A, 1353 Copenhagen, Denmark; 2 Copenhagen Affective Disorder Research Centre (CADIC), Psychiatric Centre Copenhagen, Mental Health Services, Capital Region of Denmark, Frederiksberg, Denmark; 3 Institute of Clinical Medicine, University of Copenhagen, Copenhagen, Denmark

**Keywords:** Childhood trauma, Cognition, Psychosocial functioning, Bipolar disorder

## Abstract

**Background:**

Childhood trauma (CT) are frequently reported by patients with bipolar disorder (BD), but it is unclear whether and how CT contribute to patients’ cognitive and psychosocial impairments. We aimed to examine the impact of CT on cognition and psychosocial functioning in a large sample of 345 patients with BD and 183 healthy control participants (HC) using the Childhood Trauma Questionnaire, neurocognitive tests and ratings of mood symptoms and functioning.

**Results:**

Patients showed broad cognitive impairments across memory, attention and executive function and functional disability despite being in partial or full remission and had higher levels of CT than HC. Higher levels of CT correlated with impairments across almost all cognitive domains and lower psychosocial functioning across BD patients and HC. Of these, the associations between CT and poorer working memory and lower psychosocial functioning, respectively, prevailed after adjusting for clinical and demographical variables. Diagnosis of BD and estimated verbal intelligence did not moderate these associations. Analysis of CT sub-categories showed that working memory impairments were related particularly to childhood physical and emotional *abuse,* while psychosocial difficulties were related to physical and emotional *neglect*.

**Conclusions:**

CT may have negative implications for working memory and psychosocial functioning across both BD and healthy populations. If the findings are replicated, this would suggest that early interventions that reduce the frequency of CT in vulnerable families may aid children’s cognitive and psychosocial development.

**Supplementary Information:**

The online version contains supplementary material available at 10.1186/s40345-023-00311-w.

## Introduction

Cognitive impairments during partial or full remission occur in 50–70% of patients with bipolar disorder (BD) (Burdick et al. 2014; Jensen et al. 2016; Kjærstad et al. 2021, Martínez-Arán et al. 2004a) and involve multiple domains, including attention, processing speed, verbal learning and memory, and executive functions (Bortolato et al. 2015). Cognitive impairments contribute to patients’ reduced psychosocial functioning (Sanchez-Moreno et al. 2018), such as difficulty with maintaining a job, interpersonal problems, reduced ability to live independently, and financial issues (Iosifescu 2012; Rosa et al. 2007).

Research into the origins of cognitive impairments in BD has largely focused on biological mechanisms, such as hypothalamus-pituitary-axis (HPA) dysfunction, immune dysregulation and reduced neuroplasticity (Kessing and Miskowiak 2018; Knorr et al. 2022; Porter and Gallagher 2006; Rosenblat 2015). However, emerging evidence indicates that stressful environmental factors such as childhood trauma (CT) may also play an important role (Jiménez et al. 2017; Poletti et al. 2017). Indeed, overwhelming traumatic experiences such as abuse induce a cascade of stress-mediated effects on hormones and neurotransmitters with negative effects on for the evolving brain architecture, especially within vulnerable regions, like the hippocampus and prefrontal cortex (PFC) (McCrory et al. 2010). This is noteworthy because of the high frequency of CT in individuals with BD, with studies reporting a doubling of the CT incidence in BD compared to healthy control (HC) populations (Palmier-Claus et al. 2016).

Only a few studies have so far investigated the association between CT, cognition and functioning in patients with mood disorders or schizophrenia (Aas et al. 2012; Farias et al. 2019; Jiménez et al. 2017; Kaczmarczyk et al. 2018). These studies revealed consistent associations between CT and patients’ impairments in cognition and psychosocial functioning (Aas et al. 2012; Farias et al. 2019; Jiménez et al. 2017; Kaczmarczyk et al. 2018), which may be attenuated by high premorbid intelligence (Jiménez et al. 2017). Specifically, emotional and physical abuse and emotional neglect were related to more cognitive deficits, particularly in working memory and executive function, and poorer psychosocial functioning (Aas et al. 2012; Larsson et al. 2013). Such associations between CT and impaired cognitive and psychosocial functioning have also been observed in healthy samples, indicating that this relation may be of a more *general* character (Beilharz et al. 2020; Dunn et al. 2016). However, only three published studies investigated this question directly through inclusion of both BD and HC groups (Bücker et al. 2013; Poletti et al. 2017; Savitz et al. 2008). The first study revealed a general association between CT and impaired memory and executive function across all individuals (Savitz et al. 2008); The second study found that CT was associated with executive and non-verbal memory impairments in BD but with verbal memory difficulties in HC (Bücker et al. 2013); Finally, the third study showed impaired psychomotor speed in BD patients with high (but not low) CT, whereas the opposite pattern was found in HC (Poletti et al. 2017). The conflicting findings likely result from differences in clinical characteristics of the samples and/or limited statistical power due to their small-to-moderate sample sizes (BD: n = 64–76; HC: n = 28–65).

In the present study, we investigated the association between history of CT and cognitive and psychosocial functioning a large sample of > 500 symptomatically stable BD patients and HC. We hypothesised that: (I) higher levels of CT would correlate with more impairments in cognition and psychosocial functioning across all participants, (II) individuals with high CT would display worse cognitive performance and psychosocial functioning compared to individuals with low CT, irrespective of diagnostic group, (III) CT would have more negative impact on cognition and psychosocial in BD patients than HC, in line with the diatheses-stress model, and (IV) verbal intelligence would moderate the relation between CT and cognition and psychosocial functioning, respectively, such that cognition in individuals with higher estimated verbal intelligence would be less affected by CT. The associations between specific sub-types of CT with cognition and psychosocial functioning were investigated exploratively.

## Methods

### Study design

This study is a cross-sectional examination of baseline data from patients with BD and HC pooled from three studies: (i) the Bipolar Illness Onset study (Kessing et al. 2017), (ii) the Prefrontal Target Engagement as a biomarker model for Cognitive improvement—Erythropoietin (PRETEC-EPO) study (Petersen et al. 2018), and (iii) the Prefrontal Target Engagement as a biomarker model for Cognitive improvement—Action-Based Cognitive Remediation (PRETEC-ABC) study (Ott et al. 2018). All studies have been approved by the Danish Research Ethics Committee for the Capital Region of Denmark (PRETEC-EPO: H-16043370; PRETEC-ABC: H-16043480; BIO: H-7-2014-007) and the research was carried out in accordance with the standards by the committee. Written informed consent was collected for all participants.

### Participants

Patients with BD were recruited primarily from psychiatric centres in the Capital Region of Denmark, as well as through websites. Inclusion criteria for patients were: an ICD-10 diagnosis of BD verified with the Schedules for Clinical Assessment in Neuropsychiatry (SCAN) (Wing et al. 1990), full or partial remission on the day of assessment (total scores of ≤ 7 or ≤ 14, respectively, on the Hamilton Depression Rating Scale 17-items (HDRS-17) (Hamilton 1960) or Young Mania Rating Scale (YMRS) (Young et al. 1978), fluent in Danish language and aged between 18 and 65. An additional inclusion criterion for PRETEC-ABC and PRETEC-EPO was objective cognitive impairment [total scores < 77 or below the cut-offs for cognitive impairment on ≥ 2 subtests on the Screen for Cognitive Impairment in Psychiatry (SCIP) (Jensen et al. 2015)].

Age and sex matched HC were recruited from the blood bank at Copenhagen University Hospital, Rigshospitalet. HC were excluded if they had a history of having a treatment-requiring psychiatric disorder and/or substance abuse or if they had a first-degree relative with a treatment-requiring psychiatric disorder and/or substance abuse. Exclusion criteria for all participants were severe somatic illnesses, current substance abuse and a history of brain injury or neurological illnesses, including dementia.

## Measures

### Neuropsychological test battery

Participants were assessed with a large neuropsychological test battery, including the Rey Auditory Verbal Learning Test (RAVLT) (Corwin 1994; Rey 1958), the Trail Making Test-A (TMT-A) and the Trail Making Test-B (TMT-B) (Army Individual Test Battery, 1944), the Coding and Digit Span Forward tests from the Repeatable Battery for the Assessment of Neuropsychological Status (RBANS) (Randolph et al. 1998), the Letter-Number-Sequencing subtest from Wechsler’s Adult Intelligence Scale 3rd edition (WAIS-III) (Wechsler and Psychological Corporation 1997), verbal fluency test with letters S and D (Borkowski et al. 1967) and the Spatial Working Memory (SWM) test, the One Touch Stockings of Cambridge test (OTS) and the Rapid Visual Information Processing (RVP) test from the Cambridge Neuropsychological Test Automated Battery (CANTAB). The National Adult Reading Task, Danish translation (DART) was used to estimate premorbid verbal IQ (Nelson and O’Connell 1978).

### Psychosocial functioning

Functioning was assessed with Functioning Assessment Short Test (FAST), which examines impairment in six different areas of functioning; autonomy, occupational functioning, cognitive functioning, financial issues, interpersonal relationships, and leisure time (Rosa et al. 2007).

### Childhood trauma

Participants’ history of childhood trauma was assessed with the Childhood Trauma Questionnaire (CTQ), a retrospective scale with five subscales of CT consisting of sexual, physical and emotional abuse as well as physical and emotional neglect (Bernstein et al. 2003). The CTQ consists of 28 statements describing CT, in which the participant rates the frequency using a five-point Likert Scale ranging from ‘never’ = 1 to ‘very often’ = 5. An example of a statement is “I had to wear dirty clothes”. A total score on CTQ ranges from 5 to 125, reflecting the severity of overall childhood trauma exposure (Bücker et al. 2013). When treating CT as a dichotomous low/high variable, we used a predefined cut-off score suggested by Aas et al. (2012) of ≥ 10 for physical abuse, ≥ 8 for sexual abuse, ≥  13 for emotional abuse, ≥ 15 for emotional neglect, and ≥ 10 for physical neglect. Participant were considered as having high CT when ≥ 1 subscale was above these cut-offs.

### Statistical analyses

All statistical procedures were performed using SPSS version 25. Statistical significance was set at p ≤ 0.05 (two-tailed). Shapiro–Wilk test was applied to explore data normality distribution. Nonparametric Mann–Whitney *U* tests were conducted to assess differences in demographic and clinical characteristics, cognition, psychosocial functioning as well as childhood trauma between patients and HC. We decided a priori to not adjust the analyses for multiple comparisons because of the exploratory nature of the study.

Patients’ raw scores on neuropsychological tasks were standardized to z-scale scores based on mean and standard deviation of healthy controls. Z-scores of different tests were summed and averaged to create six cognitive composites (see Additional file 1: Table S1).

To investigate the impact of CT on cognition and psychosocial functioning and whether this effect was moderated by diagnosis, we used two complementary statistical analysis approaches based on the literature: (I) Treating CT (total scores) as a *continuous* variable, correlational analyses were conducted to investigate relationships between CT, cognitive domains as well as psychosocial functioning. Furthermore, correlational analyses were conducted to investigate relationships between the different outcome variables (cognition, functioning) and the following covariates: diagnostic group, sex, age, verbal IQ, and mood symptoms. All correlational analyses were performed with Spearman’s correlations because data was not normally distributed. Based on this, multiple linear regression analyses were conducted with cognitive domains or psychosocial functioning as the dependent variable, respectively, and CT, diagnosis, the interaction between CT and diagnosis (to test hypothesis III), age, verbal IQ and the variables showing significant correlations with cognition/functioning as covariates. Age and verbal IQ were included in the regression models despite possible intercorrelation, because of the a priori hypothesis that verbal IQ may moderate the association between CT and cognitive impairment and because age in previous studies has been associated with cognitive decline (Deary et al. 2009). If mood symptoms (e.g., HDRS and YMRS) were significantly intercorrelated, only HDRS scores would be included as a covariate. (II) Treating CT as a *dichotomous* variable (low/high based on cut-off score), analyses of covariance (ANCOVA) were conducted, comparing scores on cognitive domains and psychosocial functioning for participants with high vs. low CT, controlling for sex, age, mood symptoms, verbal IQ and diagnosis (BD/HC). Significant models were followed up by post-hoc analyses in the patient sample, including total number of episodes and antipsychotic drug use as covariates.

To investigate the question of whether verbal IQ moderates the effect of CT on cognition and functioning, respectively, linear regression analyses were conducted with the cognitive domains or functioning, respectively, as the outcome variable and CT (continuous variable), the interaction between CT and verbal IQ, as well as significant covariates as predictor variables.

Finally, for cognitive domains and functioning that significantly predicted by CT (continuous variable), additional exploratory multiple linear regression analyses were conducted to investigate the associations between variables and the subtypes of CT.

## Results

### Demographics and clinical characteristics

The pooled sample included a total of 528 participants, consisting of 345 BD patients and 183 HC. Patients and HC were matched for gender and verbal IQ (ps ≥ 0.10), albeit patients were older and had fewer years of education than HC (ps < 0.003; Table 1). Expectedly, patients exhibited more subsyndromal depression and mania symptoms than HC (ps < 0.001; Table 1). Patients displayed impaired global cognitive function with a moderate effect size (r = − 0.380), as well as cognitive domain scores with small to moderate effect sizes (rs = − 0.201 to − 0.344), and more psychosocial impairments with a large effect size (r = − 0.762) compared with HC (ps ≤ 0.001; Table 1). Finally, patients had a higher total score on CTQ as well as higher scores for all subtypes of CT (ps ≤ 0.001; Table 1) and there was a higher proportion of patients than HC who were identified with ‘high CT’ (48% vs. 11%; p < 0.001). Cognition and psychosocial functioning in participants with high vs. low childhood trauma.

**Table 1:** Demographic and clinical characteristics, cognition, psychosocial functioning and childhood trauma in patients with BD (n = 345) and HC (n = 183)

	BD (n = 345)	HC (n = 183)	P-value
Gender (F/M %)	362.9/37.1	61.2/38.8	0.70
Age in years, median (IQR)	31 (16)	28 (12)	0.01*
Educational years, median (IQR)	15 (5)^1^	16 (4)	< 0.01**
Verbal IQ, median (IQR)	112.2 (7.1)^2^	112.2 (7.5)^3^	0.10
HDRS, median (IQR)	5 (6)^4^	0 (2)	< 0.01**
YMRS, median (IQR)	2 (4)^5^	0 (1)	< 0.01**
BD type, I/II %	36.5/63.5		
Illness duration in years, median (IQR)	**5** (**11**)^8^		
Number of depressive episodes, median (IQR)	**5** (**8**)^7^		
Number of (hypo)manic episodes, median (IQR)	**4** (9)^8^		
Number of mixed episodes, median (IQR)	**0** (**0**)^9^		
Total number of episodes, median (IQR)	**12 (17)**^**10**^		
Psychotropic medications			
Antidepressants, no. (%)	20.6		
Antipsychotics, no. (%)	38.6		
Anticonvulsants, no. (%)	42.9		
Lithium, no. (%)	45.8		
No medications, no. (%)	0.3		
Cognitive functions			
Global cognition, median (IQR)	− 0.46 (0.86)	0.01 (0.78)	< 0.001**
Attention, median (IQR)	− 0.47 (0.95)	0.08 (0.73)	< 0.001**
Psychomotor speed, median (IQR)	− 0.73 (1.26)	− 0.13 (1.26)	< 0.001**
Working memory, median (IQR)	− 0.55 (1.27)	0.07 (0.97)	< 0.001**
Verbal fluency and executive function, median (IQR)	− 0.39 (1.04)	0.03 (1.04)	< 0.001**
Verbal learning and memory, median (IQR)	− 0.23 (1.37)^1**1**^	0.14 (1.06)	< 0.001**
Functioning			
FAST total score, median (IQR)	18 (19)^1**2**^	0 (2)^1**3**^	< 0.001**
Childhood trauma			
CTQ total score, median (IQR)	38 (17)	29 (6)	< 0.001**
Physical abuse, total score, median (IQR)	5 (0)	5 (0)	< 0.001**
Emotional abuse, total score, median (IQR)	9 (7)	5 (1)	< 0.001**
Sexual abuse, total score, median (IQR)	5 (0)	5 (0)	< 0.001**
Emotional neglect total score, median (IQR)	11 (8)	6 (2)	< 0.001**
Physical neglect total score, median (IQR)	7 (4)	6 (4)	0.04*
High childhood trauma %	48	11	< 0.001**

Data missing for: ^1^1 participant, ^2^12 participants (not estimated primarily due to dyslexia) ^3^4 participants, ^4^1 participant, ^5^1 participant, ^6^1 participant, ^7^7 participants, ^8^6 participants, ^9^11 participants, ^10^16 participants ^11^2 participants ^12^1 participant, ^13^2 participants

*BD* Bipolar disorder, *CTQ* Childhood Trauma Questionnaire, *FAST* Functioning Short Assessment Test, *HC* Healthy Controls, *HDRS* Hamilton Depression Rating Scale, *IQR* interquartile range, *M* mean, *YMRS* Young Mania Rating Scale

^*^p ≤ 0.05, **p < 0.001

### Are higher levels of CT associated with impairments in cognition and functioning across all participants? (hypothesis I)

More childhood trauma (higher CTQ total scores) correlated mildly with poorer global cognition, attention, psychomotor speed, working memory, verbal fluency, and executive function, and moderately with more psychosocial impairments (*p*s < 0.01; Additional file 1: Table S2) but not with verbal learning and memory (p = 0.06; Additional file 1: Table S2). These significant associations prevailed for working memory and psychosocial functioning when adjusting for diagnostic group, age, sex, verbal IQ and HDRS scores (ps ≤ 0.05; see Table 2). In the patient sample, the significant association between childhood trauma and working memory prevailed when adding total number of episodes and antipsychotic drug use (*r* = − 0.12, *p* = 0.04) as covariates to the model (in addition to age, sex, verbal IQ, and HDRS scores). However, the association between childhood trauma and psychosocial impairments was reduced to near significance (*r* = 0.10, *p* = 0.08).

**Table 2:** Multiple regression investigating the association between CT (CTQ total score), CT*group, cognitive function and psychosocial functioning controlling for possible confounding effects of diagnostic group, sex, age, verbal IQ and depressive symptoms

	B	95% CI	S.E	t	p
Global cognition					
CT	− 0.00	− 0.01–0.00	0.00	− 0.88	0.38
Diagnostic group	0.32	− 0.21–− 0.86	0.27	1.19	0.24
Diagnostic group *CT	0.00	− 0.02–0.02	0.01	0.28	0.78
Age	− 0.03	− 0.04–− 0.03	0.00	− 11.85	> 0.001**
Verbal IQ	0.04	0.03–0.05	0.01	8.63	> 0.001**
HDRS	− 0.01	− 0.02–0.01	0.01	− 1.10	0.27
Attention					
CT	0.00	− 0.01–0.01	0.00	− 0.06	0.95
Diagnostic group	0.68	0.01–1.34	0.34	2.00	0.046*
Diagnostic group*CT	− 0.01	− 0.03–0.01	0.01	− 0.77	0.44
Sex	0.22	0.09–0.35	0.07	3.27	0.001*
Age	− 0.03	− 0.03–− 0.02	0.00	− 8.33	< 0.001**
Verbal IQ	0.04	0.03–0.05	0.01	6.32	< 0.001**
HDRS	− 0.01	− 0.03–0.01	0.01	− 1.12	0.26
Psychomotor speed					
CT	− 0.01	− 0.01–0.00	0.00	− 1.19	0.23
Diagnostic group	1.09	0.21–1.97	0.45	2.44	0.015*
Diagnostic group*CT	− 0.02	− 0.05–0.01	0.01	− 1.44	0.15
Sex	− 0.23	− 0.40–− 0.06	0.09	− 2.59	0.01*
Age	− 0.03	− 0.04–− 0.02	0.00	− 7.03	< 0.001**
Verbal IQ	0.03	0.02–0.05	0.01	4.16	< 0.001**
HDRS	− 0.02	− 0.04–0.01	0.01	− 1.43	0.15
Working memory					
CT	− 0.01	− 0.01–− 0.00	0.00	− 1.97	0.050*
Diagnostic group	− 0.11	− 0.83–0.61	0.37	− 0.31	0.76
Diagnostic group *CT	0.02	− 0.01–0.04	0.01	1.53	0.13
Sex	0.36	0.22–0.50	0.07	4.95	< 0.001**
Age	− 0.04	− 0.04–− 0.03	0.00	− 10.17	< 0.001**
Verbal IQ	0.04	0.03–0.05	0.01	6.52	< 0.001**
HDRS	− 0.00	− 0.02–0.02	0.01	− 0.32	0.75
Verbal fluency and executive function					
CT	0.00	− 0.00–0.01	0.00	0.69	0.49
Diagnostic group	0.27	− 0.40–0.94	0.34	0.79	0.43
Diagnostic group *CT	0.00	− 0.02–0.02	0.01	0.20	0.84
Age	− 0.02	− 0.03–− 0.02	0.00	− 7.26	< 0.001**
Verbal IQ	0.06	0.05–0.07	0.01	9.74	< 0.001**
HDRS	− 0.01	− 0.03–0.01	0.01	− 0.77	0.44
Psychosocial functioning (FAST Total score)					
CT	0.12	0.05–0.20	0.04	3.07	0.002*
Diagnostic group	− 10.65	− 17.43–− 3.87	3.45	− 3.09	0.002*
Diagnostic group*CT	− 0.11	− 0.31–0.10	0.10	− 1.04	0.30
Sex	− 3.82	− 5.59–− 2.06	0.90	− 4.25	< 0.001**
Age	0.17	0.09–0.25	0.04	4.20	< 0.001**
HDRS	0.73	0.47–0.98	0.13	5.62	< 0.001**

*CI* Confidence interval, *CTQ* Childhood Trauma Questionnaire, *FAST* Functioning Short Assessment Test, *HDRS* Hamilton Depression Rating Scale, *IQ* Intelligence Quotient, *S.E.* Standard error

^*^p ≤ 0.05, **p < 0.01

### Do individuals with high CT display worse cognition and psychosocial functioning than individuals with low CT? (hypothesis II)

There were no significant differences between participants with high and low CT in any of the cognitive domains when adjusting for diagnostic group, sex, age, HDRS, and verbal IQ (ps ≥ 0.17). However, participants with high CT had significantly poorer functioning (higher scores on FAST scores) compared to participants with low CT when controlling for diagnostic group, sex, age, HDRS, YMRS and verbal IQ (adjusted mean difference = − 2.38, 95% CI [− 4.29, − 0.47], p = 0.02). In the patient group, adding total number of episodes and antipsychotic drug use to the model, yielded a near-significant group difference between patients with high and low CT (*t* = − 1.75, *p* = 0.08).

### Does CT have more negative impact on cognition and psychosocial in BD patients than in HC? (hypothesis III)

For the multiple regression models, HDRS but not YMRS scores were included as a covariate because of high intercorrelation between HDRS and YMRS scores [r (525) = 0.43, p < 0.001; Additional file 1: Table S2]. The overall model for working memory was significant (p < 0.001) and explained 30.4% of the variance. CT, sex, age, and verbal IQ were significant predictors (CT: B = − 0.06, p = 0.050, sex: B = 0.36, p < 0.001, age: B = − 0.04, p < 0.001, verbal IQ: B = 0.04, p < 0.001). The remaining predictors including CT*diagnostic group were not significant (ps ≥ 0.13; Table 2). The remaining cognitive domains were not significantly predicted by CT (Table 2).

The overall model for FAST was significant (p < 0.001) and explained 51.4% of the variance. CT, diagnostic group, sex, age and HDRS were significant predictors (CT: B = 0.12, p = 0.002, group: B = − 10.65, p = 0.002, sex: B = − 3.82, p < 0.001, age: B = 0.17, p < 0.001, HDRS: B = 0.73, p < 0.001). The remaining predictor CT*diagnostic group was not significant (p = 0.30).

### Does verbal intelligence moderate the relation between CT, cognition, and psychosocial functioning? (hypothesis IV)

Working memory was the only cognitive domain included in this analysis because working memory was the only cognitive domain predicted by CT. The overall model for working memory was significant (p < 0.001) and explained 30.4% of the variance. Group, sex, age and verbal IQ were significant predictors (group: B = 0.41, p < 0.001, sex: B = 0.35, p < 0.001, age: B = − 0.04, p < 0.001, verbal IQ: B = 0.07, p < 0.001). The remaining predictors including verbal IQ*CTQ were not significant, ps ≥ 0.154 (Table 3).

**Table 3:** Regression investigating the association between CT, the interaction between CT and verbal IQ, and working memory, controlling for effects of diagnostic group, sex, age, and depressive symptoms

	B	95% CI	S.E	t	p
CTQ total score	0.07	− 0.03–0.17	0.05	1.33	0.18
Diagnostic group	0.42	− 0.24–0.59	0.09	4.70	< 0.001**
Verbal IQ*CTQ	− 0.00	− 0.00–0.00	0.00	− 1.43	0.15
Sex	0.35	0.20–0.49	0.07	4.79	< 0.001**
Age	− 0.04	− 0.04–− 0.03	0.00	− 10.11	< 0.001**
Verbal IQ	0.07	− 0.03–0.10	0.02	3.53	< 0.001**
HDRS	− 0.01	− 0.06–0.02	0.01	− 0.43	0.67

*CI* Confidence interval, *CTQ* Childhood Trauma Questionnaire, *HDRS* Hamilton Depression Rating Scale, *IQ* Intelligence Quotient, *S.E.* Standard error

^*^p ≤ 0.05, **p < 0.01

### Which subtypes of childhood trauma are associated with working memory and psychosocial functioning? (exploratory analyses)

The overall model for working memory with physical abuse as a predictor was significant (p < 0.001) and explained 30.4% of the variance. Physical abuse was a significant predictor (B = − 0.05, p = 0.03). Group, sex, age and verbal IQ were also significant predictors (ps ≤ 0.001), while HDRS was not (Additional file 1: Table S3). The overall model for working memory with emotional abuse as a predictor was also significant (p < 0.001) and explained 30.4% of the variance. Emotional abuse was a significant predictor (B = − 0.02, p = 0.021). Diagnostic group, sex, age, and verbal IQ were also significant predictors (ps ≤ 0.001), while HDRS was not (Additional file 1: Table S3). However, physical and emotional abuse did not predict working memory in the patient sample alone, when adding adjustment for total number of episodes and antipsychotic drug use to the model (*p*s ≥ 0.13).

The overall model for FAST with emotional neglect as a predictor was significant (p < 0.001) and explained 51% of the variance. Emotional neglect was a significant predictor (B = 0.23, p = 0.03). Group, sex, age and HDRS were also significant predictors (ps ≤ 0.001) (Additional file 1: Table S3). The overall model for FAST with physical neglect as a predictor was significant (p < 0.001) and explained 51.6% of the variance. Physical neglect was a significant predictor (B = 0.49, p = 0.001). Diagnostic group, sex, age, and HDRS were also significant predictors, (ps ≤ 0.001) (Additional file 1: Table S3). However, physical and emotional neglect did not predict FAST scores in the patient sample alone, when adding adjustment for total number of episodes and antipsychotic drug use to the model (*p*s ≥ 0.15).

## Discussion

This is the largest study to date to investigate the relation between CT, cognition, and psychosocial functioning across 345 BD patients in partial or full remission and 183 HC. We demonstrate that more CT correlate with poorer global cognitive cognition, attention, psychomotor speed, working memory, verbal fluency, and executive function, and with poorer psychosocial function *across* both BD patients and HC. Of these, the associations between CT and working memory and psychosocial function, respectively, prevailed after adjusting for demographic and clinical variables. In contrast with our hypothesis, verbal intelligence did not moderate the association between CT and impairments in working memory or psychosocial function. The subtypes of CT associated with poorer working memory were emotional and physical *abuse*, while emotional and physical *neglect* predicted lower psychosocial functioning.

The relation between CT and psychosocial functioning was robust, with a moderate association in this study. This is in keeping with other studies that found BD patients with CT to have substantially worse psychosocial functioning compared to BD patients without CT (Farias et al. 2019; Larsson et al. 2013; Sala et al. 2014). In contrast with these prior studies, the current study also involved a HC group, which enabled us to show that impact of CT on psychosocial functioning is a more *general* phenomenon that occurs across both BD and HC individuals. Our observation that physical and emotional neglect were particularly associated with poor functioning corroborates with the findings of Larsson et al. (2013). These subtypes of CT could be more frequent in families with low socioeconomic status, because these families may not have the economic means to sustain a safe home environment (Zheng et al. 2021). In line with this interpretation, *intergenerational mobility* (i.e., the relationship between socio-economic status of parents and the status their offspring attain as adults) could be a mechanism behind the relation between CT and poor functioning. Indeed, children growing up in low socioeconomic status families have an increased risk of CT as well as themselves ending in low socioeconomic status as adults (Torche 2015; Zheng et al. 2021), which may explain the observed association.

While more CT correlated with poorer performance across almost all cognitive domains, it was the association with working memory that prevailed after adjustment for demographic and clinical variables. This specific association between CT and working memory is noteworthy and consistent with previous findings by Bücker et al. (2013) and Aas et al. (2012). Working memory enables the individual to contain a smaller amount of information active for a limited time while also being able to process and manipulate this information (Baddeley 1995). Working memory is therefore crucial for comprehension, planning, reasoning, and problem solving (Cowan 2014). Long-term elevation of cortisol levels and hypothalamic pituitary adrenal (HPA) axis dysfunction during childhood could be a potential underlying mechanism by CT results in poorer working memory. Specifically, exposure to overwhelming stress triggered by CT,  including physical and emotional abuse in particular, as observed in this study, can trigger long-term elevation of cortisol levels with negative impact on the developing brain architecture (Jiang et al. 2019) and has been linked to cognitive impairments in multiple aspects of cognition, including working memory (de Souza-Talarico et al. 2011; Lupien et al. 2007; Porter and Gallagher 2006). In this way, chronically elevated cortisol and aberrant neurodevelopment may mediate the association between CT and cognitive impairments in adulthood. In keeping with this, CT has been associated with brain alterations, in vulnerable areas with prolonged developmental trajectories such as the prefrontal cortex (Pechtel and Pizzagalli 2011) and white matter integrity (Jørgensen et al., in review) which are critically important for working memory (Chai et al. 2018).

The finding that patients with BD showed no more susceptibility to CT within working memory or functioning than HC contrasts with the diathesis-stress model of psychopathology and findings by Poletti et al. (2017) in a smaller sample. Our finding suggests that BD and CT may act as *separate* risk factors for impairments in working memory and functioning and, consequently, that the negative impact of CT on these measures is of a more general character. This is in line with previous observations that CT is associated with cognitive impairments and poor psychosocial functioning also in non-clinical populations (Beilharz et al. 2020; Dunn et al. 2016; Su et al. 2019). It was also unexpected that participants with higher verbal IQ showed no more resilience to CT in working memory of functioning than participants with lower verbal IQ. This contrasts with the suggestion by, Jiménez et al. (2017) that IQ can act as a protective factor against the effect of CT on cognitive functions. However, to our knowledge, our study is the first to *directly* examine whether the relation between CT and cognition is moderated by verbal IQ through interaction effects between CT and verbal IQ in linear regression analyses. Nevertheless, further studies are warranted to examine whether our finding is replicable.

From a clinical perspective, the observed associations between CT on long-term cognitive and psychosocial outcome provide hypothesis-generating evidence that, if replicated, can  have implications for early prophylactic interventions for children at heightened risk of poor developmental trajectories. Specifically, the findings suggest a need for early interventions for at-risk children in families with psychiatric disorder to prevent CT, which may improve their cognitive development and psychosocial functioning long-term. Further, investigation of early interventions to reduce the long-term negative effects of CT, e.g., by applying eye movement desensitization and reprocessing (EMDR) or other psychotherapies for posttraumatic stress, deserve full attention.

The large sample (n = 528) was a strength of the study because it provided adequate statistical power for inclusion of multiple relevant covariates in the linear regression analyses. Further, the inclusion of a HC group enabled investigation of the generalisability of the associations between CT, cognition, and functioning. However, it could be considered a limitation that some findings did not prevail in the patient sample alone when correcting for total number of mood episodes and antipsychotic drug use. A limitation was also that we did not corrections for multiple comparisons, which was decided a priori given the explorative nature of the study. The findings should therefore be regarded as hypothesis-generating and interpreted with caution. Another limitation was the cross-sectional design, which impedes conclusions regarding causation. Further, the assessment of CT relied upon CTQ, a self-report measure with retrospective assessments of CT, which could be affected by recall bias (MacDonald et al. 2016). However, the CTQ has been shown to be a reliable and valid measure of CT (Bernstein et al. 2003). Furthermore, the CTQ does not inquire about the specific timing of traumatic events (Bernstein et al. 2003), which is a limitation since the timing of CT seems to influence their cognitive impact (Dunn et al. 2016).

In conclusion, CT was associated with poorer working memory and functioning across patients with BD and HC participants, indicating a *general* negative impact of CT beyond clinical populations. Specifically, childhood *abuse* was associated with working memory impairments, while *neglect* was associated with poorer psychosocial functioning. This points to differential causal mechanisms in cognitive impairments and psychosocial difficulties in BD. Subject to replication, the findings provide the impetus for early interventions in at-risk children to reduce CT and, thereby, aid cognitive and psychosocial outcomes long-term.

### Supplementary Information

**Additional file 1:  Table S1.** Cognitive domains composition. **Table S2.** Correlation matrix between cognitive performance, FAST, CTQ and demographic and clinical variables. **Table S3.** Multiple regression for subtypes of CT predicting working memory and functioning controlling for effects of group, sex, age, verbal IQ and depressive symptoms.

## Data Availability

The datasets used and/or analysed during the current study are available from the corresponding author on reasonable request.
