# Supplementary material for: Association between childhood trauma, cognition, and psychosocial function in a large sample of partially or fully remitted patients with bipolar disorder and healthy participants
Source: Int J Bipolar Disord. 2023 Sep 20;11:31. doi: 10.1186/s40345-023-00311-w (PMC10511386; doi:10.1186/s40345-023-00311-w)
Supplement: Supplementary file 1 — Additional file 1: Table S1. Cognitive domains composition. Table S2. Correlation matrix between cognitive performance, FAST, CTQ and demographic and clinical variables. Table S3. Multiple regression for subtypes of CT predicting working memory and functioning controlling for effects of group, sex, age, verbal IQ and depressive symptoms. [file 40345_2023_311_MOESM1_ESM.docx]

**Table S1:** Cognitive domains composition.

|  | |
| --- | --- |
| Cognitive domains | Neuropsychological tests |
| Attention | Composite of TMT-A  RBANS Digit span forward  RVP A  RVP latency |
| Psychomotor speed | Composite of SCIP (psychomotor speed)  RBANS Coding  Simple reaction time (mean correct latency) |
| Working memory | Composite of SCIP (working memory)  WAIS (Letter-Number-Sequencing)  SWM (between errors)  SWM (strategy) |
| Verbal fluency and executive function | Composite of SCIP (verbal fluency)  Verbal fluency S and D  TMT-B  OTS mean choices to correct  OTS mean latency to correct |
| Verbal learning and memory | Composite of SCIP (verbal learning and delayed recall) (VLT-I, VLT-D)) or composite of RAVLT: Trial I to V correct Trial IV  Delayed Recall  Recognition |
| Global cognitive composite | Composite of the individual cognitive domains (attention, psychomotor speed, working memory, verbal fluency and executive function, verbal learning and memory) |

**Table S2.** Correlation matrix between cognitive performance, FAST, CTQ and demographic and clinical variables

|  | | | | | | |
| --- | --- | --- | --- | --- | --- | --- |
|  |  | Age | HDRS | YMRS | Verbal IQ | CTQ total |
| **Cognition** |  |  |  |  |  |  |
| Global composite | *r*_s_ | -.36^**^ | -.23^**^ | -.13^**^ | .21^**^ | -.25^**^ |
|  | *p* | **<0.001** | **<0.001** | **0.002** | **<0.001** | **<0.001** |
|  | N | 528 | 527 | 527 | 512 | 528 |
| Attention | *r*_s_ | ^-.20**^ | -.22^**^ | -.09^*^ | .20^**^ | -.24^**^ |
|  | *p* | **<0.001** | **<0.001** | **0.049** | **<0.001** | **<0.001** |
|  | N | 528 | 527 | 527 | 512 | 528 |
| Psychomotor speed | *r*_s_ | -.26^**^ | -.19^**^ | -.10^*^ | .10^*^ | -.23^**^ |
|  | *p* | **<0.001** | **<0.001** | **0.02** | **0.03** | **<0.001** |
|  | N | 528 | 527 | 527 | 512 | 528 |
| Working memory | *r*_s_ | -.28^**^ | -.23^**^ | -.11^*^ | .17^**^ | -.25^**^ |
|  | *p* | **<0.001** | **<0.001** | **0.013** | **<0.001** | **<0.001** |
|  | N | 528 | 527 | 527 | 512 | 528 |
| Verbal fluency and executive function | *r*_s_ | -.15^**^ | -.16^**^ | -.13^**^ | .33^**^ | -.13^**^ |
|  | *p* | **0.001** | **<0.001** | **0.002** | **<0.001** | **0.002** |
|  | N | 528 | 527 | 527 | 512 | 528 |
| Verbal learning and memory | *r*_s_ | -.37** | -.10^*^ | -.10^*^ | .08 | -.08 |
|  | *p* | **<0.001** | **0.03** | **0.03** | 0.06 | 0.06 |
|  | N | 526 | 525 | 525 | 512 | 526 |
| **Psychosocial functioning** |  |  |  |  |  |  |
| FAST total | *r*_s_ | .17^**^ | .60^**^ | .29^**^ | -.05 | .48^**^ |
|  | *p* | **<0.001** | **<0.001** | **<0.001** | 0.28 | **<0.001** |
|  | N | 525 | 524 | 524 | 509 | 525 |
| **Childhood trauma** |  |  |  |  |  |  |
| CTQ total | *r*_s_ | .08 | .40^**^ | .21^**^ | -.10^*^ | 1.00 |
|  | *p* | 0.06 | **<0.001** | **<0.001** | **0.02** |  |
|  | N | 528 | 527 | 527 | 512 | 528 |
| **Demographics and clinical variables** | | | | | | |
| Age | *r*_s_ | 1.00 | .004 | -.02 | .33^**^ | .08 |
|  | *p* |  | 0.92 | 0.73 | **<0.001** | 0.06 |
|  | N | 528 | 527 | 527 | 512 | 528 |
| HDRS-17 | *r*_s_ | 0.004 | 1.000 | .43^**^ | -.08 | .40^**^ |
|  | *p* | 0.93 |  | **<0.001** | 0.08 | **<0.001** |
|  | N | 527 | 527 | 527 | 511 | 527 |
| YMRS | *r*_s_ | -.02 | .43^**^ | 1.00 | -.11^*^ | .21^**^ |
|  | *p* | 0.72 | **<0.001** |  | **0.02** | **<0.001** |
|  | N | 527 | 527 | 527 | 511 | 527 |
| Verbal IQ | *r*_s_ | .33^**^ | -.08 | -.11^*^ | 1.00 | -.10^*^ |
|  | *p* | **<0.001** | 0.08 | **0.02** |  | **0.02** |
|  | N | 512 | 511 | 511 | 512 | 512 |

*Note*. Bold text indicates significant values, *p≤0.05, **p<0.01. CTQ: Childhood Trauma Questionnaire, FAST: Functioning Short Assessment Test, HDRS: Hamilton Depression Rating Scale, *r*_s_=Spearman’s rho correlation, YMRS: Young Mania Rating Scale.

**Table S3.** Multiple regression for subtypes of CT predicting working memory and functioning controlling for effects of group, sex, age, verbal IQ and depressive symptoms.

|  | | | | | |
| --- | --- | --- | --- | --- | --- |
|  | B | 95 % CI | S.E. | t | p |
| **Working memory** |  |  |  |  |  |
| Physical abuse | -0.05 | -0.10 – -0.01 | 0.02 | -2.23 | **0.03*** |
| Diagnostic group | 0.45 | 0.29 – 0.62 | 0.08 | 5.35 | **<0.001**** |
| Sex | 0.37 | 0.23 – 0.51 | 0.07 | 5.12 | **<0.001**** |
| Age | -0.03 | -0.04 – -0.03 | 0.00 | -9.96 | **<0.001**** |
| Verbal IQ | 0.04 | 0.03 – 0.05 | 0.01 | 6.37 | **<0.001**** |
| HDRS | -0.01 | -0.03 – 0.02 | 0.01 | -0.50 | 0.62 |
| Emotional abuse | -0.02 | -0.04 – -0.00 | 0.01 | -2.31 | **0.02*** |
| Diagnostic group | 0.41 | 0.23 – 0.58 | 0.09 | 4.58 | **<0.001**** |
| Sex | 0.35 | 0.20 – 0.49 | 0.07 | 4.78 | **<0.001**** |
| Age | -0.04 | -0.04 – -0.03 | 0.00 | -10.33 | **<0.001**** |
| Verbal IQ | 0.04 | 0.03 – 0.05 | 0.01 | 6.58 | **<0.001**** |
| HDRS | 0.00 | -0.02 – 0.02 | 0.01 | -0.26 | 0.80 |
| **Psychosocial functioning (FAST total)** | | | | | |
| Emotional neglect | 0.23 | 0.03 – 0.42 | 0.10 | 2.24 | **0.03*** |
| Diagnostic group | -14.08 | -16.28 – -11.88 | 1.12 | -12.56 | **<0.001**** |
| Sex | -4.04 | -5.80 – -2.28 | 0.90 | -4.51 | **<0.001**** |
| Age | 0.17 | 0.09 –0.25 | 0.04 | 4.08 | **<0.001**** |
| HDRS | 0.75 | 0.49 – 1.00 | 0.13 | 5.75 | **<0.001**** |
| Physical neglect | 0.49 | 0.21 – 0.77 | 0.14 | 3.43 | **0.001*** |
| Diagnostic group | -14.74 | -16.79 – -12.70 | 1.04 | -14.16 | **<0.001**** |
| Sex | -3.89 | -5.64 – -2.14 | 0.89 | -4.36 | **<0.001**** |
| Age | 0.18 | 0.10 – 0.26 | 0.04 | 4.42 | **<0.001**** |
| HDRS | 0.77 | 0.52 – 1.02 | 0.13 | 6.01 | **<0.001**** |

*Note*. *p≤0.05, **p<0.01. CI: Confidence interval, CTQ: Childhood Trauma Questionnaire, FAST: Functioning Short Assessment Test, HDRS: Hamilton Depression Rating Scale, IQ: Intelligence Quotient, S.E.: Standard error.
